# Modified Renshen Wumei Decoction Alleviates Intestinal Barrier Destruction in Rats with Diarrhea

**DOI:** 10.4014/jmb.2106.06037

**Published:** 2021-07-21

**Authors:** Zhiwei Guan, Qiong Zhao, Qinwan Huang, Zhonghe Zhao, Hongyun Zhou, Yuanyuan He, Shanshan Li, Shifang Wan

**Affiliations:** 1 Hospital of Chengdu University of Traditional Chinese Medicine, Chengdu 610072, P.R. China; 2 The First Affiliated Hospital of Henan University of Traditional Chinese Medicine, Zhengzhou 450000, P.R. China; 3 College of Pharmacy, Chengdu University of Traditional Chinese Medicine, Chengdu 610075, P.R. China

**Keywords:** Modified renshen wumei decoction, diarrhea, intestinal barrier, metabolomics, rats

## Abstract

Modified Renshen Wumei decoction (MRWD), a famous traditional Chinese medicine, is widely used for treating persistent diarrhea. However, as the mechanism by which MRWD regulates diarrhea remains unknown, we examined the protective effects of MRWD on intestinal barrier integrity in a diarrhea model. In total, 48 male rats were randomly distributed to four treatment groups: the blank group (CK group), model group (MC group), Medilac-Vita group (MV group) and Chinese herb group (MRWD group). After a 21-day experiment, serum and colon samples were assessed. The diarrhea index, pathological examination findings and change in D-lactate and diamine oxidase (DAO) contents illustrated that the induction of diarrhea caused intestinal injury, which was ameliorated by MV and MRWD infusion. Metabolomics analysis identified several metabolites in the serum. Some critical metabolites, such as phosphoric acid, taurine, cortisone, leukotriene B4 and calcitriol, were found to be significantly elevated by MRWD infusion. Importantly, these differences correlated with mineral absorption and metabolism and peroxisome proliferator-activated receptor (PPAR) pathways. Moreover, it significantly increased the expression levels of TLR4, MyD88 and p-NF-κB p65 proteins and the contents of IL-1 and TNF-α, while the expression levels of occludin, claudin-1 and ZO-1 proteins decreased. These deleterious effects were significantly alleviated by MV and MRWD infusion. Our findings indicate that MRWD infusion helps alleviate diarrhea, possibly by maintaining electrolyte homeostasis, improving the intestinal barrier integrity, and inhibiting the TLR4/NF-κB axis.

## Introduction

A single layer of epithelial cells lining the gut constitutes the intestinal barrier and mainly consists of enterocyte membranes and tight junctions (TJs) between differentiated enterocytes [1-3]. Intestinal barrier integrity is essential for digesting and absorbing nutrients, and maintaining the crucial physiological barrier against invasion by exogenous pathogenic microorganisms, both in humans and animals [4, 5]. However, numerous studies have indicated that persistent diarrhea with over 14 days of watery stool impairs the growth of the intestinal mucosa, thereby hampering the function of the mechanical barrier as well as ion transport, microflora composition, and immune function [6, 7]. Malnutrition, adolescence, lack of breastfeeding, infection, and inappropriate use of antibiotics are risk factors for persistent diarrhea. In addition, antibiotic misuse during persistent diarrhea episodes has also been identified as a main risk factor [8]. At present, fluid infusion, dietotherapy, antibiotics, and intestinal mucosal protector use are common therapeutic methods for ameliorating persistent diarrhea in children [9, 10].

Traditional Chinese medicine (TCM) has offered complementary and alternative therapies for treating diarrhea; these therapies have been found to improve diarrheal symptoms, with good feedback being received from patients [11, 12]. Renshen, a popular TCM, is widely used to treat many diseases in Asian countries. It exerts beneficial effects through its primary active component, polysaccharides, which has been reported to help treat diarrhea [13]. Another famous Chinese herbal formula, Wumei Pill, is useful for treating diarrhea-predominant irritable bowel syndrome (IBS-D) by improving the ratio of Bifidobacterium/Enterobacteriaceae (B/E value) and reducing the hippocampal tissue glutamate (Glu), Î³-aminobutyric acid (Î³-GABA), dopamine (DA) and 5-hydroxytryptamine (5-HT) contents [14]. Through more than two decades of clinical practice and research, we created a novel Chinese herb, called ‘modified Renshen Wumei decoction (MRWD),’ which could be an external therapeutic agent to help ameliorate symptoms of persistent diarrhea. However, to date, there is no specific information on the underlying mechanisms by which MRWD improves intestinal barrier function in a diarrhea model, and further studies are therefore needed on this intriguing topic.

Hence, our study aimed to elaborate the effects of MRWD administration on intestinal barrier function in rats with diarrhea, providing partial theoretical evidence about the efficacy of MRWD in preventing and curing diarrhea. Our findings will also have important practical implications for the use of MRWD as a therapeutic agent to ameliorate persistent diarrhea in children.

## Materials and Methods

The experimental protocol was approved by the Ethics Review Committee for Animal Experimentation of Sichuan Academy of Laboratory Animals (P202004091). All procedures were in accordance with the Guide for the Care and Use of Laboratory Animals (National Institutes of Health).

### Animal Care and Experimental Design

Specific pathogen-free (SPF)-grade male Sprague–Dawley rats (weighing 70 ± 10 g) were supplied by Dossy Experimental Animals Co., Ltd. (China). The rats were kept in an animal room at a constant temperature (25 ± 2°C) and humidity (55 ± 10%) with 12 h of light per day and allowed food and water ad libitum before the experiment. Twelve out of 48 rats were randomly distributed to the blank group (CK group). To create diarrhea models, the other 36 rats were infused with 20 ml/kg BW senna fluid and were made to swim with a fuse wound around the tail root until they were exhausted. After successful modeling, the rats were randomly distributed to the model group (MC group), Western medicine group (Medilac-Vita) (MV group) and Chinese herb group (MRWD group). All the rats were fed a basal diet in a single cage. After 14 days following the creation of the diarrhea models, the rats in the CK and MC groups were infused with 0.9% NaCl, while those in the MV and MRWD groups were infused with 0.7 g/kg BW MV and 35 g/kg BW MRWD, respectively. The infusion was given once a day for 7 days.

### Preparation of Chinese Medicines

Preparation of 30% senna fluid: In total, 300 g of senna leaves were immersed in 2,000 ml water at 90°C for 5 min, decocted for 15 min and then filtered through four layers of gauze. After concentration using a rotary evaporator and dissolution to obtain 1,000 ml solution, the solution was stored at 4°C.

Preparation of MRWD: In total, 50 g of MRWD, consisting of 8 g of raw sun-dried Renshen, 8 g of Wumei, 5 g of hawthorn, 5 g of Chinese yam, 5 g of pomegranate peel, 5 g of *Pogostemon cablin*, 5 g of tuckahoe, 5 g of lotus seed, 1 g of baked ginger and 3 g of prepared liquorice, was boiled twice in pure water. After filtration with a 200-mesh filter cloth, the solution was combined and concentrated to 1.74 g/mL. The aforementioned Chinese medicines were provided and identified by the Pharmacy Department of Chengdu University of Traditional Chinese Medicine.

### Sample Collection

At the end of the experiment, the average body weight of rat in different groups was shown in Table 1. The rats were anesthetized by intraperitoneally injecting 3.5% chloral hydrate (350 mg/kg BW). Following this, 4 ml of abdominal aortic blood was collected and centrifuged in an EDTA anticoagulant tube at 3,500 rpm for 15 min to acquire serum samples; the samples were kept at −20°C until analysis. After blood sampling, colon samples were immediately collected from the upper 4 cm of the anus. Three pieces of a 2-cm colon sample were gently flushed with 0.9% NaCl and then fixed in 4% paraformaldehyde.

### Serum Electrolyte Detection

The concentrations of Na^+^, K^+^, Ca^2+^, and Cl^−^ were determined using spectrophotometric kits in line with the manufacturer’s instructions (Nanjing Jiancheng Bioengineering Institute, China).

### Enzyme-Linked Immunosorbent Assay (ELISA)

The D-lactate, diamine oxidase (DAO), IL-1, IL-6, TNF-α, IFN-γ, calcitriol, taurine, leukotriene B4 (LTB4) and cortisone contents were determined using spectrophotometric kits in line with the manufacturer’s instructions (Nanjing Jiancheng Bioengineering Institute).

### Western Blot Analysis

Frozen colon samples (approximately 0.1 g) were homogenized in 1 ml ice-cold RIPA lysis buffer (1% Triton X100, 10% SDS, 0.15 M NaCl, 15.4 mM Tris–HCl, 0.5% deoxycholic acid, 1 mM Na orthovanadate and Roche Mini EDTA-Free Protease Inhibitor Cocktail; pH 8.0). Following this, ultrasonication was performed to break the cells. The cells were then centrifuged at 10,000 × *g* for 15 min at 4°C. The proteins in the supernatant containing 4× Laemmli sample buffer (Bio-Rad, USA) were denatured in a 98°C metal bath for 10 min. Equal amounts of samples were then subjected to SDS-PAGE, and the expression levels of toll-like receptor (TLR4), MyD88, p-NF-κB p65, NF-κB p65, occludin, claudin‐1 and ZO‐1 proteins were assessed by western blotting using the indicated antibodies. The expression level of β-actin was assessed to ensure equal protein sample loading.

### Serum Metabolomics Analysis

In total, we mixed 100 μl serum samples and 400 μl prechilled methanol (Thermo Fisher, USA) by vortexing. The samples were incubated on ice for 5 min and then centrifuged at 15,000 × *g* at 4°C for 5 min. Some supernatants were diluted to a final concentration containing 60% methanol by LC–MS-grade water (Thermo Fisher). The samples were subsequently transferred to a fresh Eppendorf tube using a 0.22-μm filter and then centrifuged at 15,000 × *g* at 4°C for 10 min. Finally, the filtrate was injected into the LC–MS/MS system for analysis.

LC–MS/MS analysis was performed by the Vanquish UHPLC System (Thermo Fisher) coupled with an Orbitrap Q Exactive HF-X mass spectrometer (Thermo Fisher). The samples were injected onto a Hypersil Gold column (100 × 2.1 mm, 1.9 μm) using a 16 min linear gradient at a flow rate of 0.2 ml/min. The eluents for the positive polarity mode were as follows: eluent A (0.1% FA in water) and eluent B (methanol). The eluents for the negative polarity mode were as follows: eluent A (5 mM ammonium acetate 153, pH 9.0) and eluent B (methanol). The solvent gradient was set as follows: 2% B, 1.5 min; 2–100% B, 12 min; 100% B, 14 min; 100–2% B, 14.1 min and 2% B, 16 min. The Q Exactive HF-X mass spectrometer was operated with a spray voltage of 3.2 kV, capillary temperature of 320°C, sheath gas flow rate of 35 arb and aux gas flow rate of 10 arb.

The raw data files generated by UHPLC–MS/MS were processed using Compound Discoverer 3.0 (CD 3.0, Thermo Fisher) for peak alignment, peak picking and quantification of each metabolite. The metabolites were annotated using the KEGG database (http://www.genome.jp/kegg/), HMDB database (http://www.hmdb.ca/) and LIPID MAPS database (http://www.lipidmaps.org/). Principal component analysis (PCA) and partial least squares discriminant analysis (PLS‐DA) were performed using metaX (a flexible and comprehensive software program for processing metabolomics data). Univariate analysis (*t*-test) was performed to determine the statistical significance (*p*-value). Metabolites with a Variable Importance in Projection (VIP) score > 1, *p*-value < 0.05 and fold change (FC) ≥ 2 or FC ≤ 0.5 were considered differential metabolites. Volcano plots were used to filter metabolites of interest based on their log_2_(FC) and −log10(*p*-value).

For clustering heat maps, the data were normalized using z-scores of the intensity areas of differential metabolites and plotted using the pheatmap package in R language. The correlation between differential metabolites was assessed using the cor() function in R language (method = Pearson’s correlation). Statistical significance of the correlation between differential metabolites was calculated using cor.mtest() in R language. *P*-values < 0.05 were considered statistically significant, and correlation plots were plotted using the corrplot package in R language. The functions of these metabolites and metabolic pathways were assessed using the KEGG database. Metabolic pathway enrichment of differential metabolites was performed. If x/n > y/N, the metabolic pathway was recognized as enriched. If the *p*-value of the metabolic pathway was < 0.05, the metabolic pathway was regarded as significantly enriched.

### Statistical Analysis

SPSS 24.0 statistical software (IBM, USA) was used to analyze all the data. If the data conformed to a normal distribution and the variance was homogeneous, the LSD method was used after one-way ANOVA for multiple comparisons. Otherwise, Tamhane’s T2 multiple comparison was adopted. The data are displayed as the means ± SE. *p*-values < 0.05 were considered statistically significant.

## Results

### Physical and Physiological Effects in the MC and MRWD Groups

**Fig. 1:**
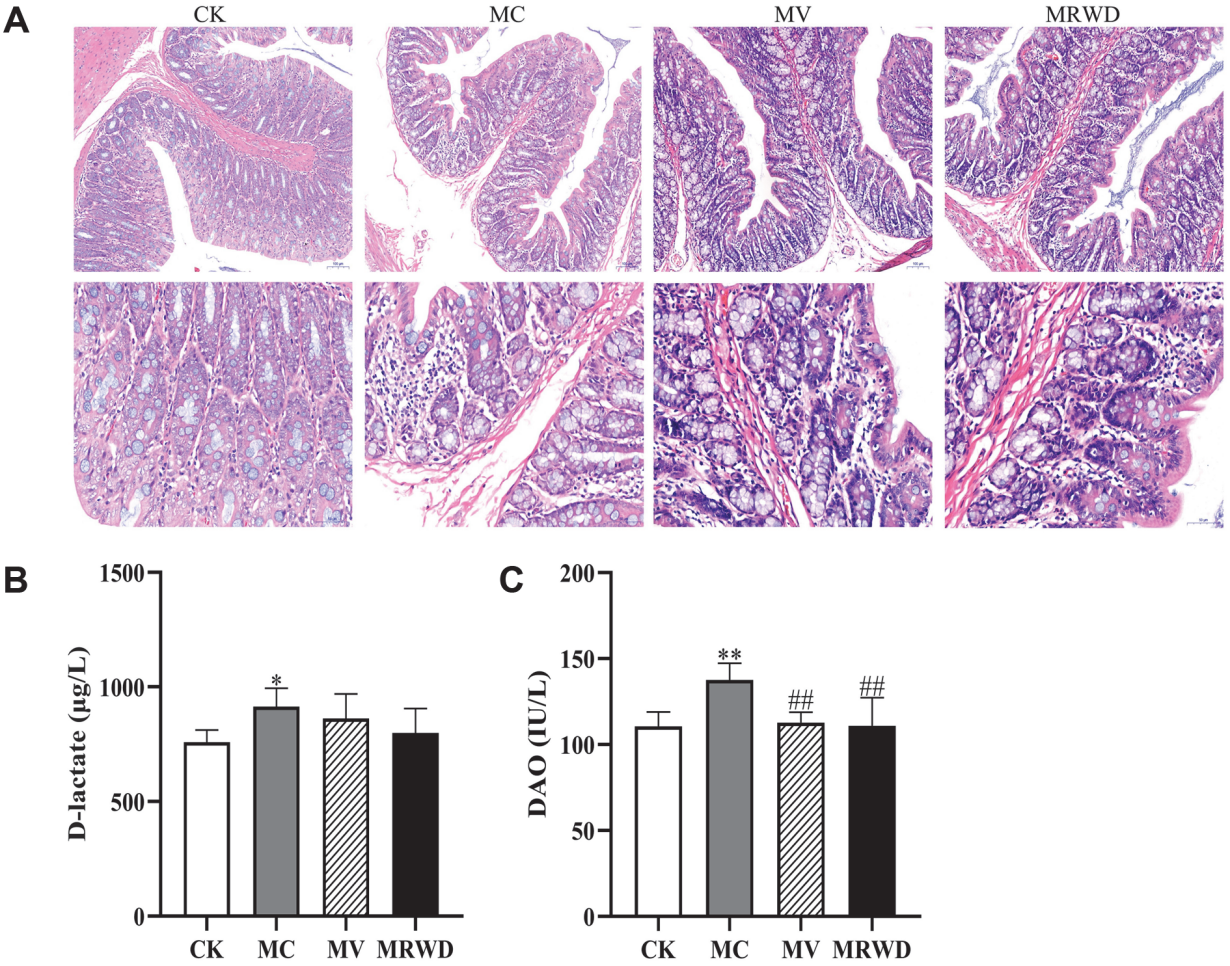
Physical and physiological effects of MRWD on the colon of rats. (**A**) Haematoxylin–eosin (HE) staining of colon tissue. (**B**) The concentration of D-lactate was detected by ELISA. (**C**) The concentration of DAO was detected by ELISA. *Compared with the CK group, *p < 0.05 and **p < 0.01. ^#^Compared with the MC group, ^#^*p* < 0.05 and ^##^*p* < 0.01.

The diarrhea assessment results revealed that the prevalence of loose stools and diarrhea index were significantly higher (*p* < 0.01) in the MC group than that in the MV and MRWD groups (Table 2). Compared with the CK group, HE staining revealed obvious pathological changes in the colon in the MC group; however, these pathological changes were lesser in the MV and MRWD groups (Fig. 1A). The contents of D-lactate and DAO, biomarkers of intestinal injury, were significantly higher in the MC group than that in the CK group (*p* < 0.05); however, the contents were lesser in the MV and MRWD groups (Figs. 1B and C). These results indicate that MRWD infusion alleviates the destruction of the intestinal morphology induced by diarrhea.

### Optimization and Validation of UHPLC–MS/MS Methods

**Fig. 2:**
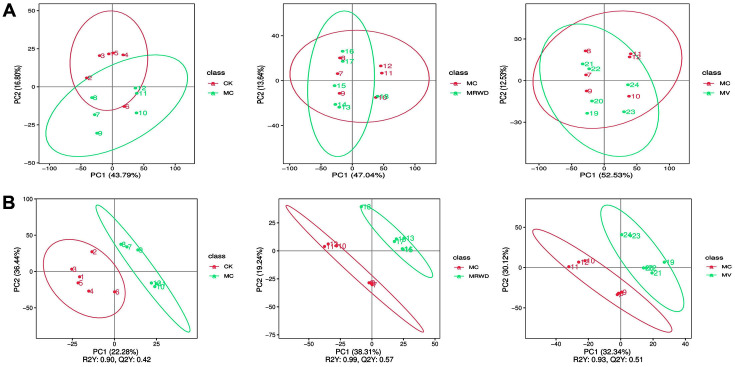
Multivariate statistical analysis plot of CK, MC MV and MRWD groups. The PCA (**A**) and PLS-DA (**B**) score plots demonstrate complete separation of the serum samples among the groups.

The differences in metabolites among the CK, MC, MV and MRWD groups were assessed by PCA. As shown in Fig. 2A, the PCA score plot provided the clustering images of each group; these images were well distinguished, indicating that MV and MRWD infusion could partly regulate the metabolic disorders caused by diarrhea. To further analyze the metabolomics model for each group and to identify the potential differential metabolites with significant changes, PLS-DA was performed. Compared with PCA, PLS-DA emphasizes the differences between groups and weakens the random differences unrelated to the purpose of the study. This enables a better understanding of the overall characteristics and variation rules of multidimensional metabolomics data, which is conducive to the discovery of intergroup differences and metabolic markers causing differences. The score plot provided in Fig. 2B shows a clear separation among the four groups of serum samples.

### Identification of Potential Metabolites in the Serum Samples of Diarrhea Model Rats Following MRWD Infusion

**Fig. 3:**
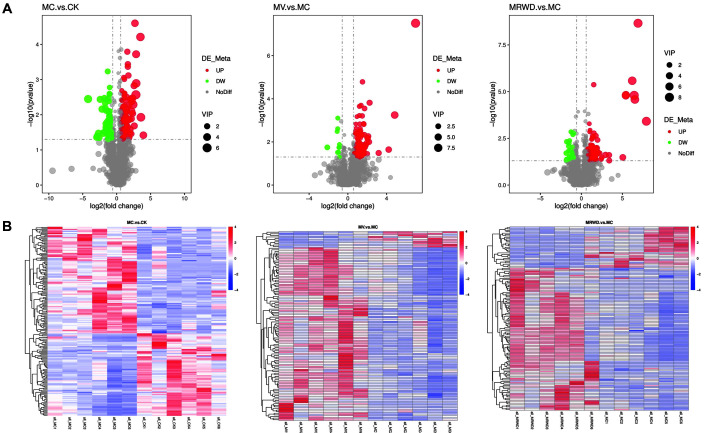
Analysis of differential metabolites in the serum samples obtained from CK, MC, MV and MRWD groups. (**A**) A volcano plot representing the significant variables for discriminating among the four groups. Significantly increased variables are presented in the red circle, while significantly decreased variables are presented in the green circle. (**B**) A heatmap of hierarchical clustering analysis (HCA) for differential metabolites. HCA is based on the Euclidean distance; colors from blue to red indicate elevated contents of metabolites.

Of the numerous compound signals detected in the CK, MC, MV and MRWD groups, variables that significantly contributed to clustering and discrimination were identified according to the following threshold: VIP score ≥ 1.0. This threshold was obtained after PLS-DA processing of the variables (Fig. 2B). Based on the VIP score, variables were selected from the four groups for univariate analysis (*t*-test). When the *p*-value was < 0.05, the compounds were screened as potential differential metabolites for identification. The differential metabolites obtained from the four groups were analyzed by one-way ANOVA, and the results without significant differences were removed. The screening results of the differential metabolites are presented as a volcano plot in Fig. 3A. Table 3 summarizes the 20 potentially differentiated metabolites obtained from the four groups and their retention times, mass charge ratios and molecular formulas. For clustering heatmaps, the data were assessed and plotted in R language. The results showed that the metabolic pattern of the differential metabolites obviously differed among the four groups (Fig. 3B).

### Analysis of Signal Pathways Using the Serum Samples of Diarrhea Model Rats Following MRWD Infusion

**Fig. 4:**
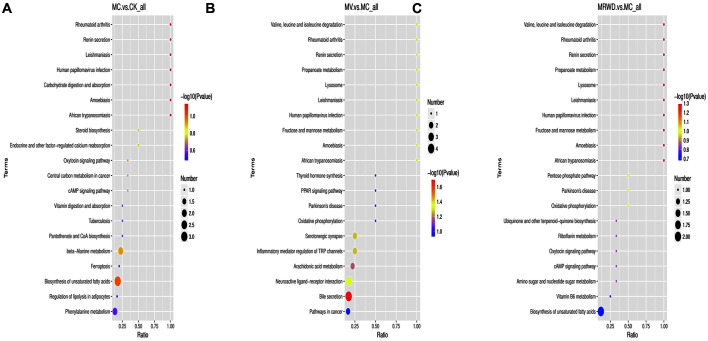
Pathway analysis of differential metabolites in the serum samples. A bubble plot for the identification of the most relevant metabolic pathways. The color of the circles indicates the significance of changes in the metabolites in the corresponding pathway, while the size corresponds to the pathway impact score. The pathway impact score represents the cumulative percentage from the matched metabolite to the total pathway importance.

The KEGG database was used to assess the relevance of the pathways to determine the locations of the differential metabolites among the groups. In fact, the bubble map of the metabolic pathway analysis indicated that dramatically disturbed metabolic pathways included mineral absorption, carbohydrate digestion and absorption, vitamin digestion and absorption, metabolic pathways, steroid biosynthesis, endocrine- and other factor-regulated calcium reabsorption, protein digestion and absorption and oxidative phosphorylation (Fig. 4).

### Validation of the Representative Metabolites

**Fig. 5:**
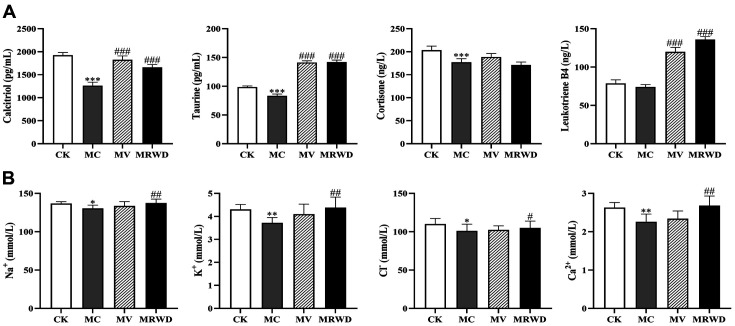
Effect of MRWD on the serum parameters. (**A**) Validation of the representative metabolites by the metabolomics approach using ELISA. (**B**) The analysis of serum electrolyte contents. *Compared with the CK group, *p < 0.05, **p < 0.01 and ***p < 0.001. ^#^Compared with the MC group, ^#^*p* < 0.05, ^##^*p* < 0.01 and ^###^*p* < 0.001.

Based on metabolomics data, metabolites, including calcitriol, taurine, LTB4 and cortisone, were screened. Following this, the effects of MRWD on the representative metabolites were assessed by ELISA. As shown in Fig. 5A, the induction of diarrhea significantly reduced the calcitriol, taurine and cortisone contents (*p* < 0.01). Compared with the MC group, the calcitriol, taurine and LTB4 contents were markedly increased in the MV and MRWD groups (*p* < 0.01).

### Analysis of Serum Electrolytes

Spectrophotometric kits were used to detect the concentrations of serum electrolytes, including Na^+^, K^+^, Ca^2+^, and Cl^−^. As shown in Fig. 5B, the induction of diarrhea significantly decreased the electrolyte concentrations (*p* < 0.05). However, the electrolyte concentrations were increased by the MRWD infusion (*p* < 0.05).

### Analysis of Intestinal Integrity

**Fig. 6:**
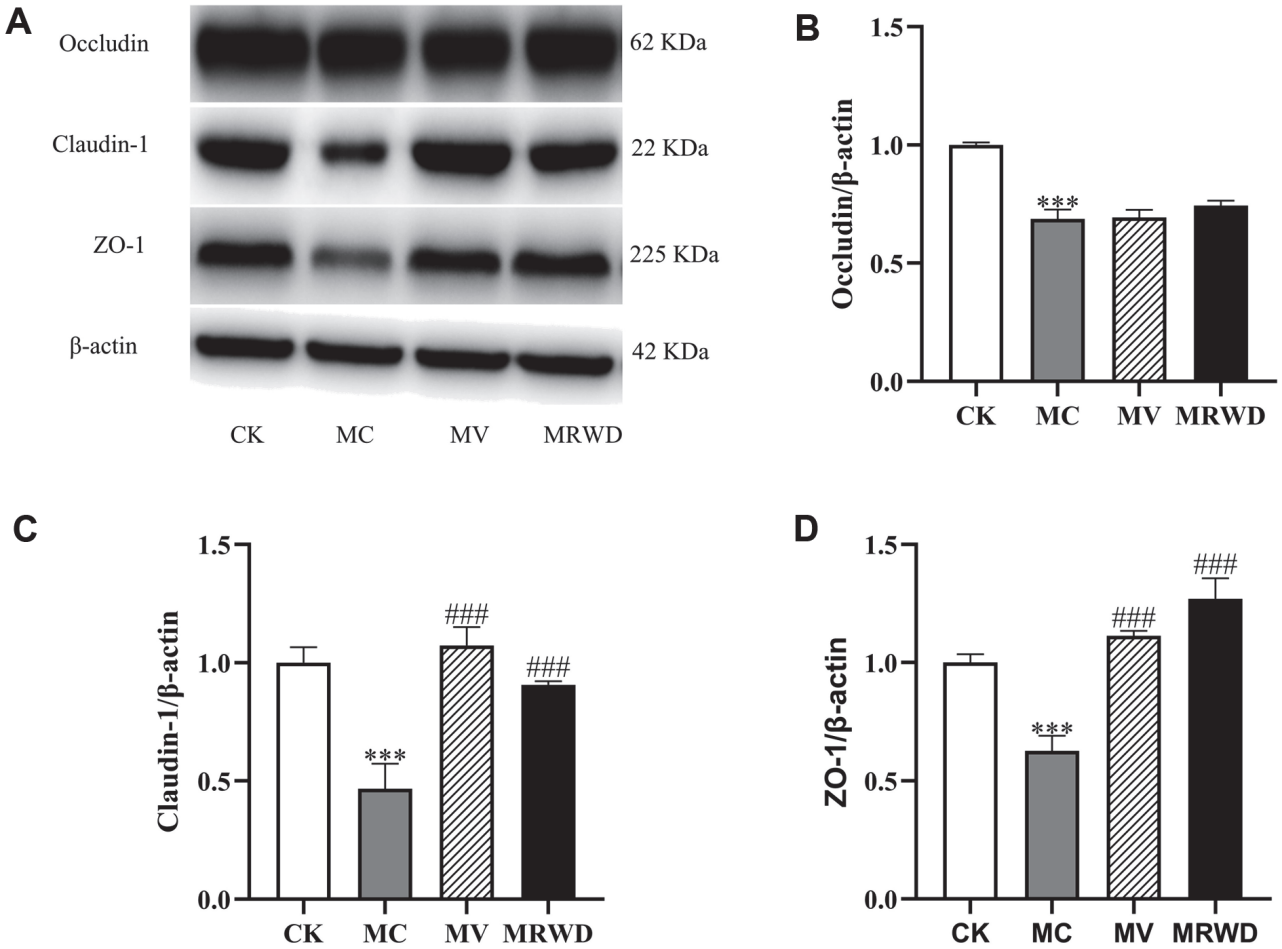
MV and MRWD infusion alleviated the destruction of tight junctions (TJs). (**A**) The colon samples were analyzed by western blotting using antibodies against claudin-1, ZO-1, occludin and β-actin (used as the protein loading control). (**B**) The occludin/β-actin ratio in the colon. (**C**) The claudin-1/β-actin ratio in the colon. (**D**) The ZO-1/β-actin ratio in the colon. *Compared with the CK group, *p < 0.05, **p < 0.01 and ***p < 0.001. ^#^Compared with the MC group, ^#^*p* < 0.05, ^##^*P* < 0.01 and ^###^*p* < 0.001.

The differences in intestinal barrier-related variables among the four groups indicated that the expression levels of occludin, claudin-1 and ZO-1 proteins were lower in the MC group (*p* < 0.01) than that in the CK group. However, MV and MRWD infusion increased (*p* < 0.05) the expression levels of claudin-1 and ZO-1 proteins (Fig. 6).

### Analysis of Inflammation-Related Parameters

**Fig. 7:**
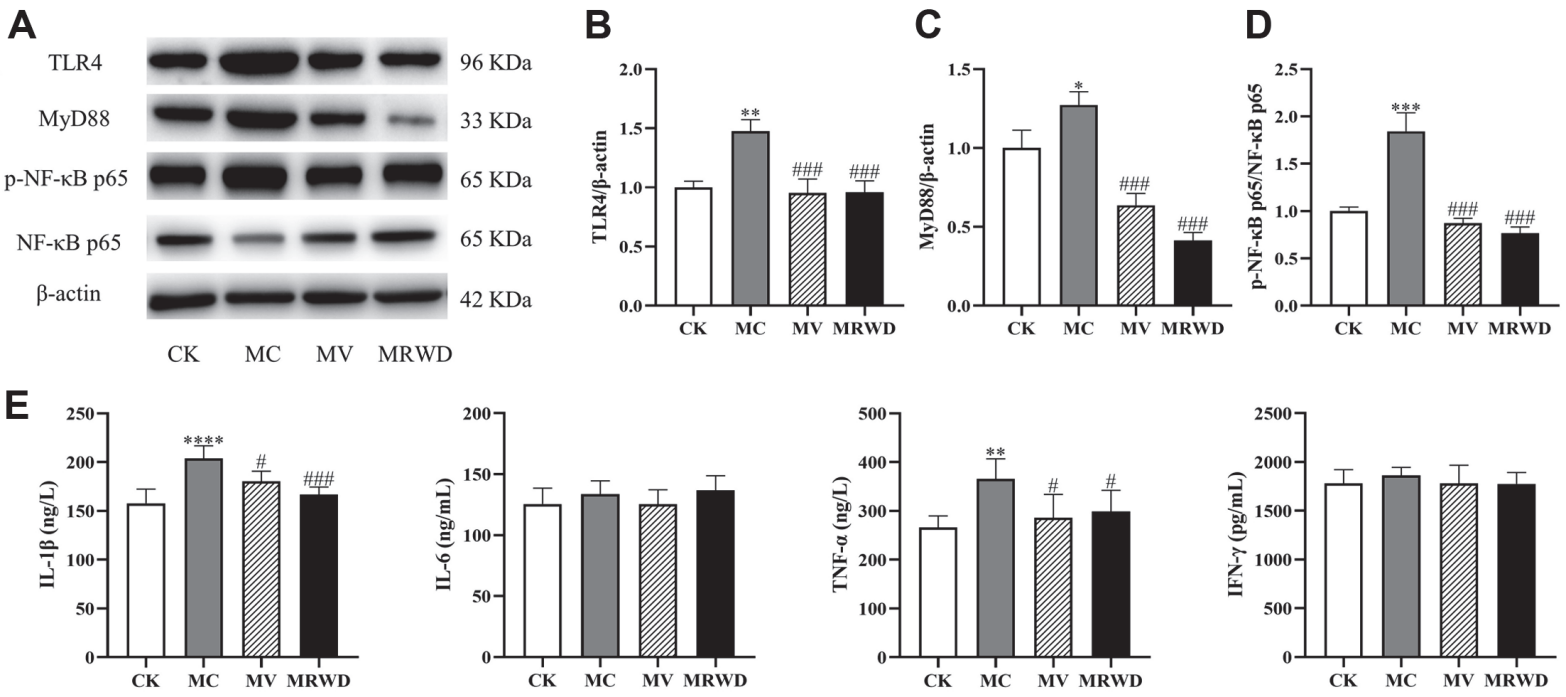
MV and MRWD infusion decreased the inflammatory response in rats with diarrhea. (**A**) The colon samples were analyzed by western blotting using antibodies against TLR4, MyD88, p-NF-κB p65, NF-κB p65 and β-actin (used as the protein loading control). (**B**) The TLR4/β-actin ratio in the colon. (**C**) The MyD88/β-actin ratio in the colon. (**D**) The p-NF-κB p65/NF-κB p65 ratio in the colon. (**E**) The IL-1, IL-6, TNF-α and IFN-γ contents were assessed by ELISA. *Compared with the CK group, *p < 0.05, **p < 0.01 and ***p < 0.001. ^#^Compared with the MC group, ^#^*p* < 0.05, ^##^*p* < 0.01 and ^###^*p* < 0.001.

As shown in Fig. 7A, the induction of diarrhea increased the expression levels of TLR4, MyD88 and p-NF-κB p65 proteins (*p* < 0.05). The expression levels of TLR4, MyD88 and p-NF-κB p65 proteins were markedly lower in the MV and MRWD groups than that in the MC group (*p* < 0.01). In addition, the IL-1 and TNF-α contents were higher in the MC group than that in the MC and MRWD groups (*p* < 0.05). Nevertheless, IL-6 and IFN-γ were not prominently influenced (*p* > 0.05) by the induction of diarrhea (Fig. 7B).

## Discussion

Maintenance of the integrity of intestinal epithelial cells (IECs) and control of the permeability between adjacent IECs are the minimum requirements to ensure the intestinal barrier functioning properly [15]. However, the intestinal function is often compromised in patients with persistent diarrhea. Consequently, maintaining normal intestinal architecture and functioning is essential for alleviating diarrhea. In our study, we noticed that MRWD obviously decreased the diarrhea index and restored the impaired intestinal architecture in rats with diarrhea. Serum D-lactic acid and DAO are two well-established markers for monitoring changes in intestinal permeability [16]. The increase in the serum D-lactate and DAO contents has been reported to correlate with the extent of intestinal barrier dysfunction [17, 18]. Consistent with this finding, we noted a decrease in the serum D-lactate and DAO contents following MRWD infusion. Moreover, TJs connect neighboring IECs and play a vital role in paracellular solute permeability [19]. The reconfiguration of TJs makes the epithelial barrier more conducive to the paracellular permeation of fluid and cations, predominantly Na^+^, which can decrease transepithelial electric resistance (TEER) [20]. Besides, it was reported that a single material of MRWD possesses the protective function of TJ proteins to alleviate the destruction in different disease models. Pomegranate peel polyphenols (PPPs) alleviated HFD-induced depressed colonic tight junction protein expression level in rats. In addition, it has been confirmed that PPPs increased the LPS-induced decreased tight junction protein expression level in Caco-2 cells [21]. Chinese yam phenanthrene 4 (CYP4) pretreatments substantially improved tight junction protein occludin in dextran sulfate sodium (DSS)-treated mice [22]. Hence, TJs and serum ion distribution were also assessed. The expression levels of occludin, claudin-1 and ZO-1 proteins and the electrolyte concentrations were markedly abrogated by the induction of diarrhea; however, this finding was not noted in the MV and MRWD groups. These results support the notion that MRWD exerts beneficial effects on the intestinal barrier integrity in rats with diarrhea.

In addition to damaging the intestinal integrity and disrupting electrolyte homeostasis in the colon of rats, the induction of diarrhea triggers intestinal inflammation marked by upregulated expression levels of pro-inflammatory cytokines in the intestine [23, 24]. Previous studies revealed that pro-inflammatory cytokines such as IL-1 and TNF-α could increase the intestinal TJ permeability, which has been postulated to be an important factor in the exacerbation of intestinal inflammation [25-27]. In our study, MV and MRWD infusion was found to decrease the IL-1 and TNF-α contents induced by diarrhea; this finding is consistent with the improved intestinal barrier integrity. The existing scientific evidence states that inflammatory responses can be regulated by various signaling pathways. Hence, we also assessed the molecular mechanisms by which MRWD affects intestinal inflammatory responses in rats with diarrhea.

TLR is a type of protein with a type I transmembrane receptor, which is rich in extracellular leucine repeat domains and intracellular transcription/IL-1 receptor domains [28]. TLR4 is the best-characterized member of the TLR family; it is activated by bacterial lipopolysaccharide (LPS), thus initiating an innate immune response and inflammation in higher animals [29, 30]. In our study, we found that the colonic expression levels of TLR4 protein and its downstream target, MyD88, were decreased in the MV and MRWD groups. A previous study demonstrated that the genetic knockout of TLR4 (*Tlr4^−/−^*) protected against the development of barrier disorder and mitigated the duration and severity of diarrhea [31]. Therefore, the downregulation of the TLR4 signaling pathway and its downstream cytokine production may help improve the intestinal integrity following MRWD infusion. At the same time, single materials of MRWD, such as pogostemon cablin, Chinese yam, ginger, and pomegranate peel, protect the host via suppressing TLR4/ NF-κB signaling and its downstream inflammatory cytokines [32-34]. Subsequently, we assessed the expression of p-NF-κB p65 protein. As expected, MV and MRWD infusion suppressed the expression level of p-NF-κB p65 protein. Therefore, we reasoned that the inhibition of intestinal pro-inflammatory cytokines was linked to the blocking of NF-κB expression via the suppression of the TLR4 signaling pathways in the MRWD group.

In the present study, serum samples were analyzed by a UHPLC–MS/MS-based metabolomics approach to detect the effect of MRWD infusion on the endogenous metabolites; significant differences in the levels of the endogenous metabolites were observed among the four groups. Studies have shown that phosphoric acid can promote intestinal maturation and the growth rate of longfin yellowtail fish by stimulating digestive enzymes [35]. KEGG enrichment results indicated that phosphoric acid plays an important role in mineral absorption as well as oxidative phosphorylation. The induction of diarrhea significantly decreased the content of phosphoric acid, while MRWD infusion alleviated this decrease, with the content in the MRWD group being higher than that in the CK group. In the present study, the calcitriol content in the MC group was found to be lower than that in the CK group. An MRWD/MC ratio greater than 1 indicated that MRWD infusion increased the calcitriol content. The altered calcitriol content indicated that intestinal calcium absorption was abrogated by the induction of diarrhea but alleviated by MRWD infusion [36]. Similarly, the taurine content was lower in the MC group than in the CK group; however, MRWD infusion increased the taurine content in rats with diarrhea. KEGG enrichment results indicated that taurine is vital to the transport of minerals and organic ions. These changes indicated that intestinal absorption was markedly hampered by the induction of diarrhea but was alleviated by MRWD infusion. Moreover, the content of LTB4, which is related to the PPAR pathway, was significantly increased by MRWD infusion. Given the damage to intestinal barrier function by inflammatory cytokines, *e.g.*, TNF-α, IL-1 and IL-6, studies have revealed that PPARs plays an anti-inflammatory role by subverting NF-κB activation; this finding is consistent with the results of western blotting and ELISA [37, 38]. In brief, the serum metabolites showed an abnormal change, which might be closely related to abnormal intestinal absorption and anti-inflammatory function. MRWD infusion could alleviate diarrhea by improving intestinal absorption and immune function.

In summary, the present findings indicate that MRWD administration can improve intestinal barrier function by promoting electrolyte transport, intestinal integrity and inhibiting the TLR4/NF-κB signaling pathways. These changes were found to be accompanied by the alleviation of diarrhea in rats. Our findings provide a scientific basis for the use of MRWD for treating persistent diarrhea.

## Figures and Tables

**Table 1:** The average body weight of rats in different treatment groups at the time of sacrifice.^a^

Groups	Average body weight (g)
CK	197.68±23.24
MC	158.24±13.33***
MV	156.60±13.47
MRWD	174.55±9.70^#^

^a^Values are means with standard deviations (*n* = 8).

*Compared with the CK group, ***p < 0.001. ^#^Compared with the MC group, ^#^*p* < 0.05.

**Table 2:** Assessment of diarrhea in rats.^a^

Groups	CK	MC	MV	MRWD
Prevalence of loose stools	/	89.46 ± 7.85	36.37 ± 4.61^##^	32.16 ± 4.31^##^
Diarrhoea index	/	2.32 ± 0.48	0.90 ± 0.16^##^	0.72 ± 0.17^##^

^a^Values are means with standard deviations (*n* = 8).

^#^Compared with the MC group, ^#^*p* < 0.05 and ^##^*p* < 0.01.

**Table 3:** Quantitative comparison of serum metabolites.

Metabolite	Formula	RT (min)	Mass	Ratio			*P -* value		

				MC/CK	MV/MC	MRWD/MC	MC/CK	MV/MC	MRWD/MC
Phosphoric acid	H_3_O_4_P	1.16	97.98	1.01	1.70	2.53	0.95	<0.01	0.02
Cortisone	C_21_H_28_O_5_	11.22	360.19	0.71	0.88	0.65	0.20	0.57	0.34
Leukotriene B4	C_20_H_32_O_4_	12.37	336.23	0.81	1.66	1.89	0.23	0.01	0.02
allophanic acid	C_2_H_4_N_2_O_3_	104.02	5.51	1.62	0.93	1.39	0.11	0.52	0.01
Calcitriol	C_27_H_44_O_3_	416.33	14.52	0.35	0.99	1.04	0.03	0.98	0.84
demethylphylloquinone	C_30_H_44_O_2_	436.33	14.04	0.82	1.16	2.90	0.18	0.39	0.05
Naphthalene	C_10_H_8_	128.06	13.34	1.48	1.66	1.50	0.33	0.13	0.27
Taurine	C_2_H_7_NO_3_S	125.01	1.40	0.94	1.48	1.53	0.65	0.02	0.02
Pantothenic acid	C_9_H_17_NO_5_	219.11	1.86	1.90	1.14	1.46	0.01	0.45	0.08
Luteolin	C_15_H_10_O_6_	286.05	9.72	7.14	0.57	0.34	0.00	0.15	0.02
Kaempferol	C_15_H_10_O_6_	286.05	7.16	4.95	0.64	0.44	0.00	0.30	0.06
D-Ribulose 5-phosphate	C_5_H_11_O_8_P	230.02	1.19	1.23	1.52	1.70	0.38	0.05	0.01
Xylitol	C_5_H_12_O_5_	152.07	1.36	1.06	0.99	0.87	0.44	0.82	0.05
3-Methylbutanoic acid	C_5_H_10_O_2_	102.07	1.59	0.45	2.45	3.63	0.26	0.13	0.01
Dihomo-gamma-linolenic acid	C_20_H_34_O_2_	306.26	13.69	0.74	0.98	2.33	0.56	0.98	0.02
Sucralose	C_12_H_19_Cl_3_O_8_	396.01	8.41	2.92	0.52	0.34	0.02	0.28	0.06
L-Histidine	C_6_H_9_N_3_O_2_	155.07	1.34	0.39	0.53	1.23	0.03	0.41	0.93
Methylmalonic acid	C_4_H_6_O_4_	118.03	1.16	0.47	1.88	2.95	0.06	<0.01	0.02
Prostaglandin E2	C_20_H_32_O_5_	352.23	11.13	0.48	3.30	2.11	<0.01	<0.01	0.02
Adrenic acid	C_22_H_36_O_2_	332.27	14.78	0.56	1.27	0.98	0.04	0.22	0.79
